# Dietary Protein Source and Litter Condition Alter Broiler Chicken Intestinal Macrophage and Mitotically Active Cell Populations

**DOI:** 10.3389/fvets.2022.894587

**Published:** 2022-04-13

**Authors:** A. Jacob Keel, Allan J. Calderon, Oscar J. Tejeda, Jessica D. Starkey, Charles W. Starkey

**Affiliations:** Department of Poultry Science, Auburn University, Auburn, AL, United States

**Keywords:** broiler chicken, intestinal innate immunity, macrophage, mitotic activity, litter conditions, dietary protein source

## Abstract

As antibiotic-free (ABF) broiler production continues to increase, understanding the development and local immune response in the intestines of ABF broilers is essential. Mitotically active cells, the majority of which will become enterocytes, help maintain the intestinal epithelial barrier. Macrophages prevent pathogen invasion by their phagocytic activity, functioning as immune response amplifying cells to aid in the recruitment of additional immune cells, and stimulating cytokine production in other adjacent cells. The objective of this experiment was to evaluate commonly used practical production practices on intestinal cell mitotic activity and local intestinal immunological responses. A randomized complete block design experiment with a 3 × 2 factorial treatment structure was conducted. The 3 dietary protein sources were: soybean meal (SBM), a mix of 50% poultry by-product meal and 50% feather meal (PFM), and porcine meat and bone meal (MBM) and broilers were reared on either new litter (NL) or used litter (UL). On d 3, 8, 11, 15, and 21, 6 birds per treatment from 6 blocks (total *n* = 36 per d) were randomly selected for sampling. Broilers were injected intraperitoneally with 5'-bromo-2'-deoxyuridine (BrdU) 1 h prior to sampling to label mitotically active cells. Samples were analyzed using cryohistology and immunofluorescence to determine the density of mitotically active cells and macrophages. Mitotically active cell and macrophage densities changed in both the duodenum and ileum over time. Neither dietary protein source nor litter condition affected mitotically active cell or macrophage densities in the duodenum on d 11 and 21 or in the ileum on d 3, 8, 11, and 15. However, on d 3 and 15 in the duodenum (*P* ≤ 0.0126) and d 21 in the ileum (*P* ≤ 0.0009), broilers reared on UL had greater mitotically active cell densities than those reared on NL. On d 8 in the duodenum, broilers fed MBM had increased macrophage density compared with those fed PFM and SBM (*P* ≤ 0.0401). These results indicate dietary protein source and litter condition may impact the physiology of the broiler small intestine, though additional work with this model is necessary to understand the underlying mechanisms.

## Introduction

The broiler industry is primarily concerned with producing broiler growth via lean muscle accretion; however, several different physiological systems support the complex process of muscle growth and development. Although necessary, these systems can place a burden on broiler metabolism and reduce the efficiency of muscle protein accretion by requiring energy and nutrients that could be utilized for lean muscle growth. It has been estimated that maintenance of the gastrointestinal tract (GIT) alone requires 25% of the total basal metabolic needs of an animal, while this tissue does not contribute as significantly as lean muscle tissue to the profit gained from broiler processing (1). However, growth and maintenance of the GIT are important for it to perform its roles of digestion and nutrient absorption, without which muscle growth would be impossible. Intestinal growth and maintenance are supported by two groups of proliferative cells, and these are intestinal stem cells (ISC) and transit amplifying (TA) cells. As ISC divide, the daughter cells enter the TA cell pool, which divide and differentiate into the mature cell types of the intestinal epithelium (2).

Intestinal epithelial cells continuously migrate up the villi and are shed upon reaching the tip. Also, the intestinal epithelium provides some immune protection as its integrity must be maintained to prevent translocation of microorganisms into host tissue. Furthermore, there is a strong presence of immune cells within the GIT, and it contains the largest pool of tissue-resident macrophages found in the body (3). This helps illustrate the vital role the GIT plays in maintaining the health of an animal, by defending against ingested pathogens and managing the growth and composition of the intestinal microbiota. Therefore, in order to continue improving the sustainability and efficiency of broiler production, it will be important to develop a better understanding of the broiler GIT in terms of its development, maintenance, and immune functions.

Currently, the broiler industry is experiencing a shift away from the use of antibiotic growth promoters (AGP). As of 2019, more than 50% of broilers produced in the United States were reared under some type of antibiotic-free (ABF) program (4). In the past, the broiler industry has relied on AGP to improve the efficiency of broiler production as research has indicated that AGP reduce intestinal length and weight and produce an anti-inflammatory effect (5–7). Thus, these two benefits of AGP help to shift the partitioning of energy and nutrients away from metabolic functions, such as GIT maintenance and immunology, that can be unfavorable to broiler production. Overall, this contributes to improved growth performance, health, and mortality of broilers receiving AGP (8).

Antibiotic-free broiler production has increased the incidence of enteric diseases like coccidiosis and necrotic enteritis (NE), both of which can induce costly production loses (9–11). Certain controllable elements of broiler management, such as dietary protein source and litter condition, may influence the occurrence of enteric disease (12, 13). The control of coccidiosis and NE, whether through exogenous treatments or by the broiler immune system itself, is important as some research has suggested that high growth rate broilers may be less adept at quickly controlling and resolving an immune challenge (14, 15). A prolonged immune response can lead to worsened feed conversion ratio (FCR), increased immune protein synthesis, and increased liver mass (16). Immune proteins differ in amino acid ratios compared with skeletal muscle proteins, and diets are formulated to meet the requirements of skeletal muscle, which may mean that during an immune response the appropriate amino acids are not provided in the diet. Thus, skeletal muscle may act as an amino acid reservoir and be catabolized to free amino acids needed for sufficient immune function (17). In the face of present challenges of ABF broiler production, it is aware that knowledge is lacking in the areas of broiler GIT development and immunology that may aid in revealing and evaluating appropriate AGP alternatives and improving broiler performance in an ABF environment. Therefore, the major objectives of this study were to establish an experimental model where 2 major GIT development and local immune parameters could be measured and simultaneously assess how dietary protein source and litter condition influence these variables in young broilers. The success of the methods and changes over time described here will aid in the future study of GIT development and health in ABF broilers.

## Materials and Methods

All procedures involving the use of live birds in this work were approved by the Auburn University Institutional Animal Care and Use Committee under protocol 2017-3146.

### Dietary and Litter Treatments

This experiment was a randomized complete block design (*n* = 50 blocks, each comprised of 6 pens, with each pen representing a different treatment) with a 3 × 2 factorial treatment arrangement. Three diets were fed, differing primarily in the dietary protein source included. The dietary protein sources were soybean meal (SBM), porcine meat and bone meal (MBM), and a 50:50% poultry by-product and feather meal mix (PFM). These diets were formulated based on diets typically fed in the broiler industry, therefore, each of the diets contained some SBM, but the MBM and PFM diets included 4% MBM or PFM, respectively (Table 1). Two different litter conditions were also used to imitate common industry practices. Broilers were either reared on new litter (NL; fresh pine shavings) or used litter (UL: pine shavings previously used by 3 flocks). The UL was obtained from the Auburn University Poultry Research Unit and was previously used in nutrition experiments, not disease trials, making it a suitable mimic of reused industry litter.

**Table 1:** Composition of starter diets fed to broilers reared to 21 d-of-age.

	**Dietary protein source treatment**		
**Ingredient, %**	**MBM^**a**^**	**PFM^**b**^**	**SBM^**c**^**
Corn	61.07	65.52	60.42
MBM^a^	4	-	-
PFM^b^	-	4	-
SBM^c^	32.29	27.79	35.54
Soybean oil	0.94	0.5	1.47
Dicalcium phosphate	0.55	0.95	1.34
Calcium carbonate	0.57	0.67	0.69
Salt	0.37	0.36	0.33
Phytase	0.01	0.01	0.01
Vitamin and mineral premix^*d*^, ^*e*^	0.2	0.2	0.2

a *MBM, porcine meat and bone meal*.

b *PFM, 50% poultry by-product and 50% feather meal*.

c *SBM, soybean meal*.

d *Vitamin premix provided the following per kilogram of diet: Vitamin A (Vitamin A acetate), 9,370 IU; Vitamin D (cholecalciferol), 3,300 IU; Vitamin E (DL-alpha tocopheryl acetate), 33 IU; menadione (menadione sodium bisulfate complex), 2 mg; Vitamin B12 (cyanocobalamin), 0.02 mg; folacin (folic acid), 1.3 mg: D-pantothenic acid (calcium pantothenate), 15 mg; riboflavin (riboflavin), 11 mg; niacin (niacinamide), 44 mg; thiamin (thiamin mononitrate), 2.7 mg; D-biotin (biotin), 0.09 mg; and pyridoxine (pyridoxine hydrochloride), 3.8 mg*.

e *Mineral premix includes per kg of diet: Mn (manganese sulfate), 120 mg; Zn (zinc sulfate), 100 mg; Fe (iron sulfate monohydrate), 30 mg; Cu (tri-basic copper chloride), 8 mg; I (stabilized ethylenediamine dihydriodide), 1.4 mg; Se (sodium selenite), 0.3 mg*.

### Bird Husbandry

On d of hatch, 1,500 Yield Plus × Ross 708 female broiler chicks (Aviagen Group, Huntsville AL) were received from a commercial hatchery. Each bird was tagged with a wing tag for identification, individually weighed to determine d 0 bodyweight, and randomly distributed to 300 pens (n = 5 birds per pen at placement; 0.04 m^2^ per bird). On d 8, each pen was culled to 3 birds per pen (0.07 m^2^ per bird). Each raised floor pen was equipped with 1 feeder and 2 water nipples, providing *ad libitum* access to feed and water. A crumbled starter diet was supplied throughout the experiment (1–21 d). At placement, ambient temperature was set at 30.5°C and reduced to maintain bird comfort as the birds aged, with a final set point of 24.5°C on d 21. From d 0–7, birds were exposed to 30 lux of light for 23 h, and from d 8 to 21, the lighting was adjusted to 18 h of light at 10 lux with 6 h of darkness. Individual bird body weights were recorded, and pen feeder weights were collected to calculate individual bodyweight gain (BWG) and mortality corrected pen feed intake (MCFI). Mortality was recorded daily, and FCR was mortality corrected (Supplementary Table 1).

### Tissue Sample Collection and Analysis

#### Bromodeoxyuridine Injection

Sample collection was performed on d 3, 8, 11, 15, and 21. On each sampling day, 6 blocks were randomly selected, and 1 bird was randomly selected from each of the pens in those blocks for sample collection (total *n* = 36 sampled per sampling day). Birds were injected intraperitoneally with an aqueous solution (25 mg per mL) of 5'-bromo-2'-deoxyuridine (BrdU; Alfa Aesar, Haverhill, MA; 100 μg of BrdU per g of body weight) to label mitotically active cells, as described by Hutton et al. (18). Following BrdU injection, birds were placed in disposable containers for 1 h to allow BrdU to be incorporated into mitotically active cells. BrdU is incorporated into the DNA of proliferating cells in S-phase of mitosis, providing a label that can be detected during later immunofluorescence microscopy analysis. After the 1 h cell-labeling period, birds were euthanized by CO_2_ asphyxiation, followed by immediate cervical dislocation.

Immediately following euthanasia, tissue samples were collected from the proximal and distal small intestine, duodenum, and ileum, respectively. The duodenal samples were collected just distal to the duodenal loop to avoid accidental inclusion of pancreatic tissue, and the ileal samples were collected proximal to the ileocecal junction. After being excised, the small intestine samples were flushed with phosphate buffered saline (PBS; pH 7.4; Invitrogen, Carlsbad, CA) to remove intestinal contents. The samples were then flash frozen in liquid nitrogen and stored at −80°C. Before freezing, the cylindrical samples were cut lengthwise to better expose the mucosal surface to the liquid nitrogen and promote rapid freezing, rather than slow freezing which would damage the morphology of the tissue.

#### Cryohistological Analysis

Samples were held at −20°C for 24 h to bring samples to an appropriate temperature for cryosectioning. After being embedded in frozen section compound (VWR International, Westchester, PA), 5-μm thick cryosections were collected from the intestinal samples, transverse to the length of the intestine, using a Leica CM 1950 cryomicrotome. The cryosections were mounted on positively charged glass slides (VWR International; 5 cryosections per slide) and stored at 4°C for no more than 48 h before immunofluorescence staining.

#### Immunofluorescence Staining

Cryosections were fixed and immunofluorescence stained following previously published methods with some modifications as described below (18–20). All procedures were conducted at room temperature. Slides were first rehydrated in PBS for 10 min and then fixed in paraformaldehyde (4% in PBS; VWR International) for 10 min, followed by 2 rinses in PBS. Next, slides were treated with 0.5% Triton X-100 (VWR International) for 10 min to permeabilize the cell membranes. Following permeabilization, slides were exposed to 1N hydrochloric acid for 10 min to denature the DNA and improve BrdU detection, followed by a single rinse in PBS. The slides were then incubated for 30 min in a blocking solution containing 10% horse serum (Sigma-Aldrich), 2% bovine serum albumin (VWR International), and 0.2% Triton X-100 in PBS to prevent non-specific antibody binding. Antibody reactions were conducted in a dark, humidified box. Primary antibodies were diluted in blocking solution and allowed to react with tissue cryosections for 1 h, followed by rinsing 3 times for 5 min per rinse in PBS. Similarly, secondary antibodies were also diluted in blocking solution and allowed to react with tissue cryosections for 30 min, followed by rinsing 3 times for 5 min per rinse in PBS. Then, the cryosections were exposed to 4', 6-diamidino-phenylindol (DAPI; 1 μg per mL; VWR International) to label all nuclei within the tissue and immediately rinsed twice in PBS. Slides were mounted using florescence mounting media with Tris buffer (Fluoro-gel, Electron Microscopy Sciences, Hatfield, PA) and thin glass coverslips (VWR International), then permanently sealed with clear fingernail polish (Sally Hansen, New York, NY) and allowed to dry at room temperature. All cryosections were digitally imaged within 72 h of immunofluorescence staining.

#### Primary and Secondary Antibodies

Primary antibodies were as follows: mouse monoclonal IgG1 anti-chicken monocyte/macrophage [KUL01, 1:500 dilution, Cat. No. SC52603, Santa Cruz Biotechnology, Santa Cruz, CA, validated by Mast et al. (21)] and mouse monoclonal IgG2a anti-BrdU (1:500 dilution, Cat. No. MA3-071, Invitrogen, Thermo Fisher Scientific Inc., Waltham, MA). Secondary antibodies (Invitrogen; 1:1000 dilution) were: Alexa-Fluor 546 goat anti-mouse IgG1 (Cat. No. A21123) and Alexa-Fluor 633 goat anti-mouse IgG2a (Cat. No. A21136).

#### Fluorescence Photomicroscopy and Digital Image Analysis

**Figure 1:**
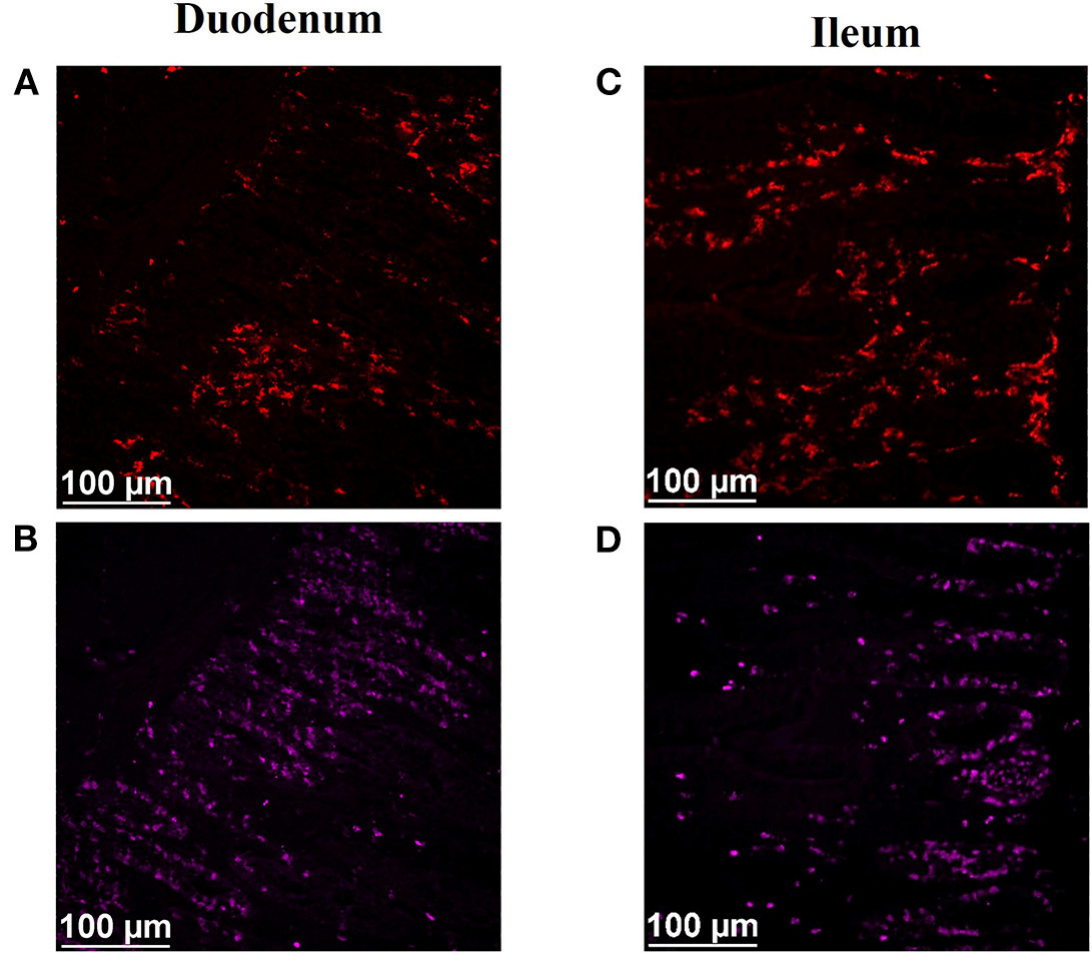
Effect of dietary protein source and litter condition on macrophage (KUL01+) and mitotically active (BrdU+) cell densities (cells per mm^2^) in the duodenum of 3-d-old broilers (*n* = 36 per sampling d, 6 birds per treatment). MBM, meat and bone meal; PFM, 50% poultry by-product and 50% feather meal blend; SBM, soybean meal; NL, new litter; UL, used litter. ^a, b^Bars of the same color representing means from either dietary protein source or litter condition with different superscripts differ at *P* ≤ 0.05.

After immunofluorescence staining, cryosections were imaged at 200X magnification using a semi-automated inverted fluorescence microscope (Nikon Eclipse Ti-U; Nikon Instruments, Inc., Melville, NY) equipped with a solid-state LED light source (Lumencor SOLA Sm II 365 light engine). Images were captured and analyzed using an Evolve 512 EMCCF camera (Photometrics, Tuscon, AZ) and the Basic Research version of Elements software (Nikon Instruments, Inc.). Two representative images were captured from each slide (1 slide per bird), focusing on the crypt region of the tissue section. The area of mucosa in each image was measured, and BrdU+ mucosal nuclei and macrophages located within the *lamina propria* were counted to calculate mitotically active cell and macrophage density, which are reported as the number of positive cells per square millimeter. All nuclei determined to be positive for BrdU were also labeled with DAPI, a nuclear counterstain. Representative digital images illustrating detection of KUL01+ macrophages, including pro-inflammatory and anti-inflammatory macrophages, and mitotically active cells in duodenal and ileal cryosections of 21-d-old broilers are shown in Figure 1.

### Statistical Analysis

All data were subjected to 2 and 3-way analysis of variance using the GLIMMIX procedure of SAS version 9.4 (SAS Institute, Cary, NC), which is suitable for both normally and non-normally distributed datasets. For the 2-way ANOVA dietary protein source and litter condition served as the fixed effects with bird as the experimental unit with 6 replicates per treatment per sampling time point. For the 3-way ANOVA, dietary protein source, litter condition, and time (bird d-of-age) served as the fixed effects to assess their impact on cell populations over time. The Satterthwaite adjustment was used to correct the degrees of freedom and the PDIFF option was used to conduct the pairwise least square means separation analysis. For all variables, the means were separated at *P* ≤ 0.05.

## Results and Discussion

The major goal of this work was to establish experimental procedures to assess GIT developmental (mitotically active cell density) and local immune (total macrophage density) responses while simultaneously determining how dietary protein source, litter condition, and time (bird age) impact these variables. The ability to successfully evaluate local cellular responses will improve our overall understanding of what is required to successfully feed and manage broilers in ABF production systems. Interestingly, no 2 or 3-way interactions among the fixed effects of dietary protein source, litter condition, and time (broiler d-of-age) were observed for either mitotically active cell density or macrophage density (*P* > 0.05). Therefore, the results are presented as fixed effects within a sampling age time point and over time by tissue (duodenum and ileum).

**Figure 2:**
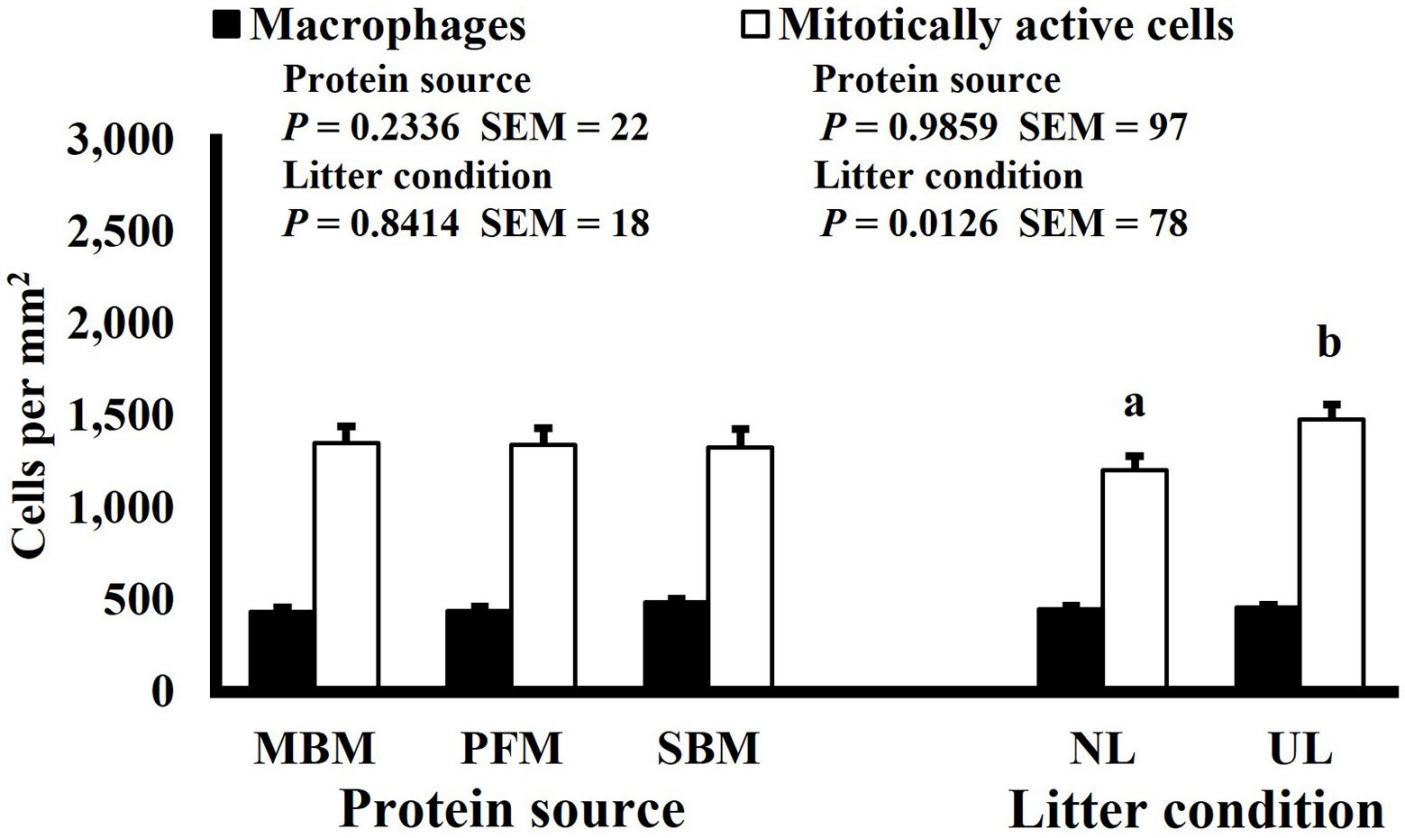
Effect of dietary protein source and litter condition on macrophage (KUL01+) and mitotically active (BrdU+) cell densities (cells per mm^2^) in the duodenum of 15-d-old broilers (*n* = 36 per sampling d, 6 birds per treatment). MBM, meat and bone meal; PFM, 50% poultry by-product and 50% feather meal blend; SBM, soybean meal; NL, new litter; UL, used litter. ^a, b^Bars of the same color representing means from either dietary protein source or litter condition with different superscripts differ at *P* ≤ 0.05.

**Figure 3:**
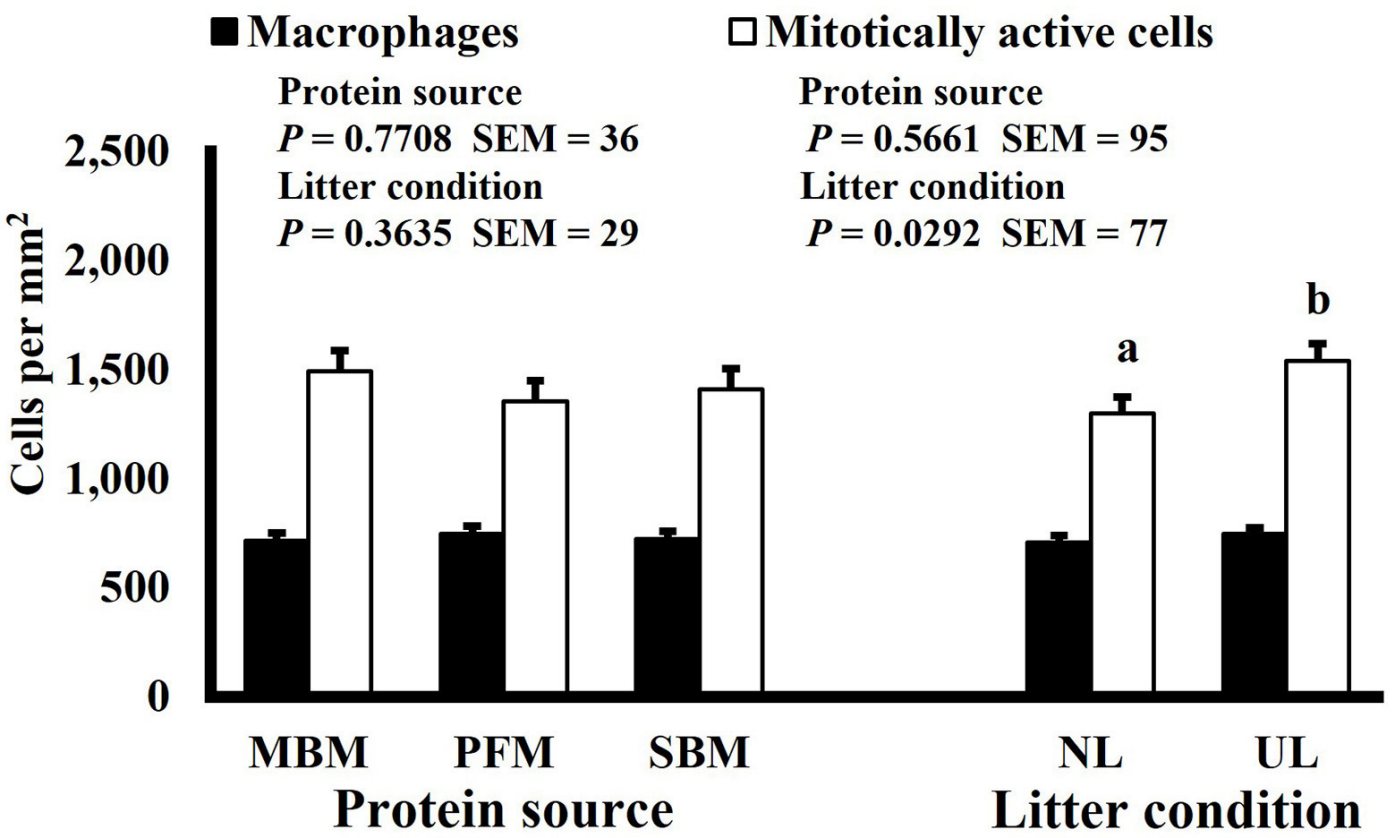
Effect of dietary protein source and litter condition on macrophage (KUL01+) and mitotically active (BrdU+) cell densities (cells per mm^2^) in the ileum of 21-d-old broilers (*n* = 36 per sampling d, 6 birds per treatment). MBM, meat and bone meal; PFM, 50% poultry by-product and 50% feather meal blend; SBM, soybean meal; NL, new litter; UL, used litter. ^a, b^Bars of the same color representing means from either dietary protein source or litter condition with different superscripts differ at *P* ≤ 0.05.

**Figure 4:**
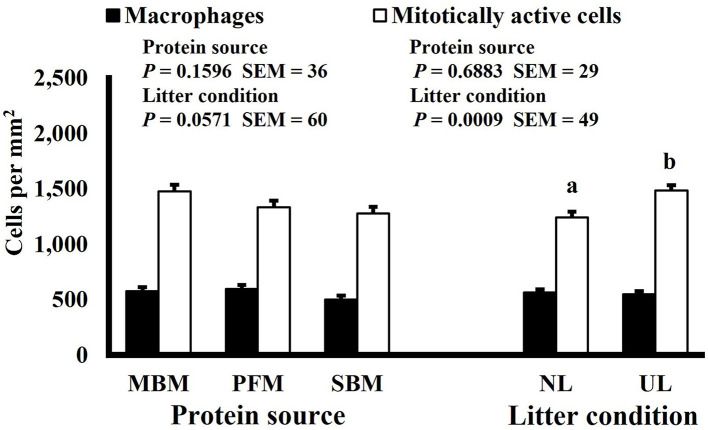
Effect of dietary protein source and litter condition on macrophage (KUL01+) and mitotically active (BrdU+) cell densities (cells per mm^2^) in the duodenum of 11-d-old broilers (*n* = 36 per sampling d, 6 birds per treatment). MBM, meat and bone meal; PFM, 50% poultry by-product and 50% feather meal blend; SBM, soybean meal; NL, new litter; UL, used litter.

Mitotic activity of GIT cells is required for maintenance and growth of intestinal villi but increases in mitotically active cell density may also be needed to replace villus cells that were damaged due to some type of insult or in response to changes in enterocyte migration rate. In this study, on d 3 and 15 (*P* ≤ 0.0126; Figures 2, 3, respectively) and d 21 in the ileum (*P* ≤ 0.0009; Figure 4), broilers reared on UL had greater duodenal mitotically active cell density than those reared on NL (1,481 vs. 1,202 on d 3 and 1,540 vs. 1,298 on d 15 in the duodenum; 1,484 vs. 1,244 on d 21 in the ileum). Chickens are often observed scratching, pecking, and consuming litter, and in the case of used litter, which can contain microorganisms shed by past flocks, litter consumption could expose chicks to pathogens or other microorganisms that they are not immunologically mature enough to manage or defend against. Previous research has indicated that UL can alter the profile of the intestinal microbiota toward more intestinal derived microbes (12). The microbiota may play a role in regulating intestinal epithelial proliferation (22). Although it cannot be definitively stated based on the parameters measured in this study, the higher density of mitotically active cells may be due to an altered intestinal microbiota in broilers reared on UL. Parker et al. suggested that under the influence of TNF-induced intestinal inflammation, murine villi decrease in size due to apoptosis of enterocytes of the villus body but a change in proliferation rate was not observed (23). However, changes in proliferation rate during the resolution of inflammation were not studied, and changes may occur during this time to return the tissue to its homeostatic state.

**Figure 5:**
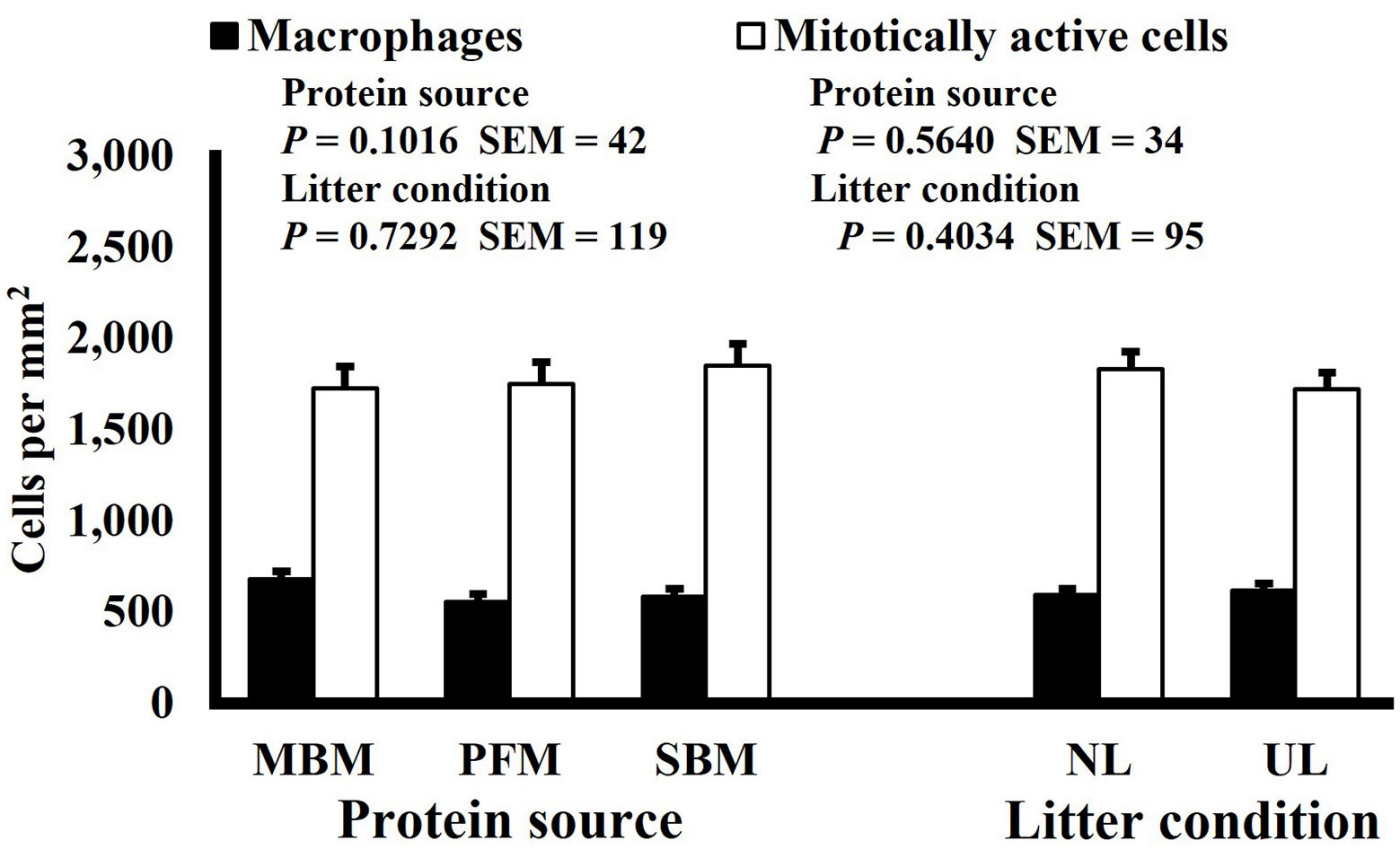
Effect of dietary protein source and litter condition on macrophage (KUL01+) and mitotically active (BrdU+) cell densities (cells per mm^2^) in the duodenum of 21-d-old broilers (*n* = 36 per sampling d, 6 birds per treatment). MBM, meat and bone meal; PFM, 50% poultry by-product and 50% feather meal blend; SBM, soybean meal; NL, new litter; UL, used litter.

**Figure 6:**
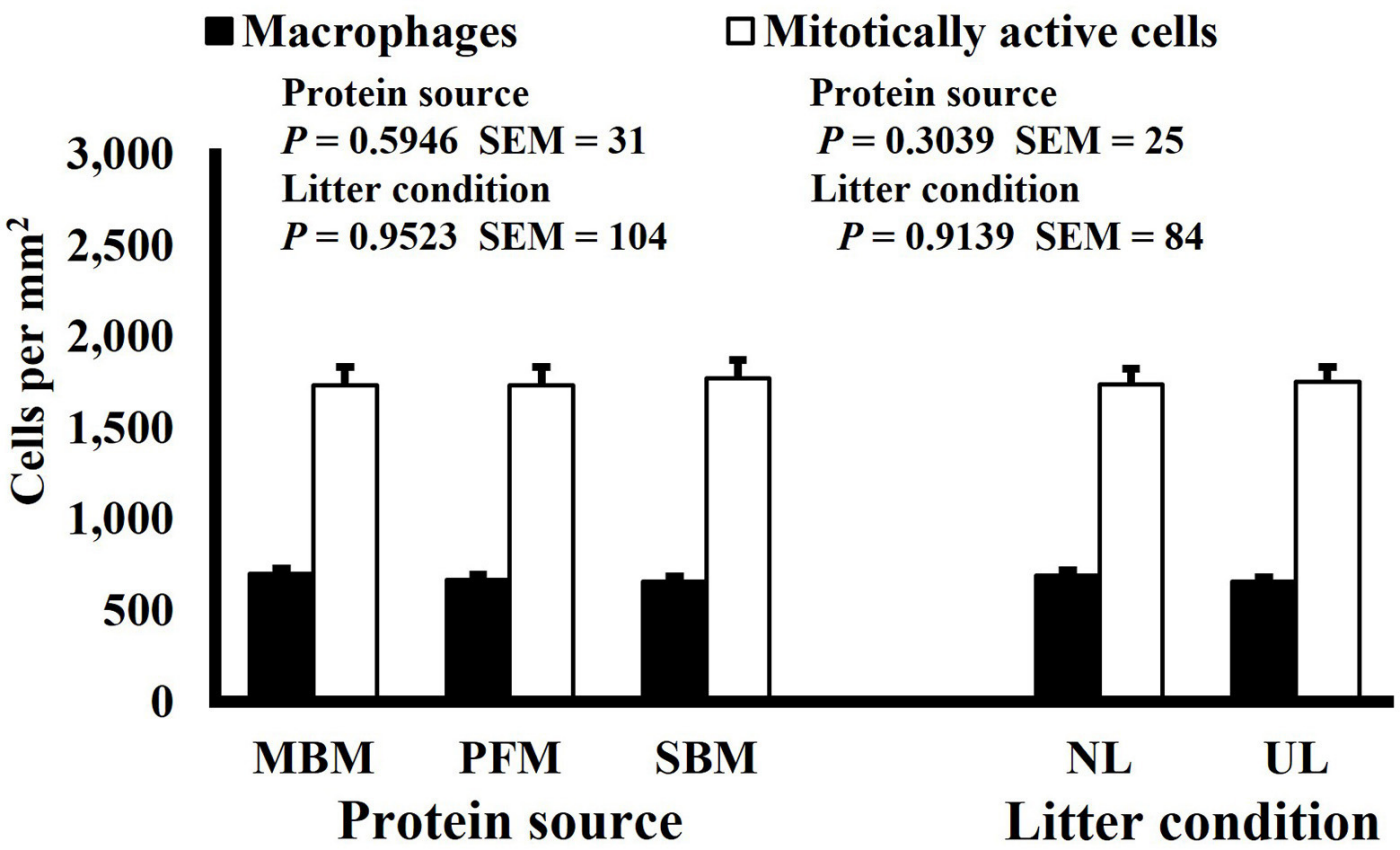
Effect of dietary protein source and litter condition on macrophage (KUL01+) and mitotically active (BrdU+) cell densities (cells per mm^2^) in the ileum of 3-d-old broilers (*n* = 36 per sampling d, 6 birds per treatment). MBM, meat and bone meal; PFM, 50% poultry by-product and 50% feather meal blend; SBM, soybean meal; NL, new litter; UL, used litter.

**Figure 7:**
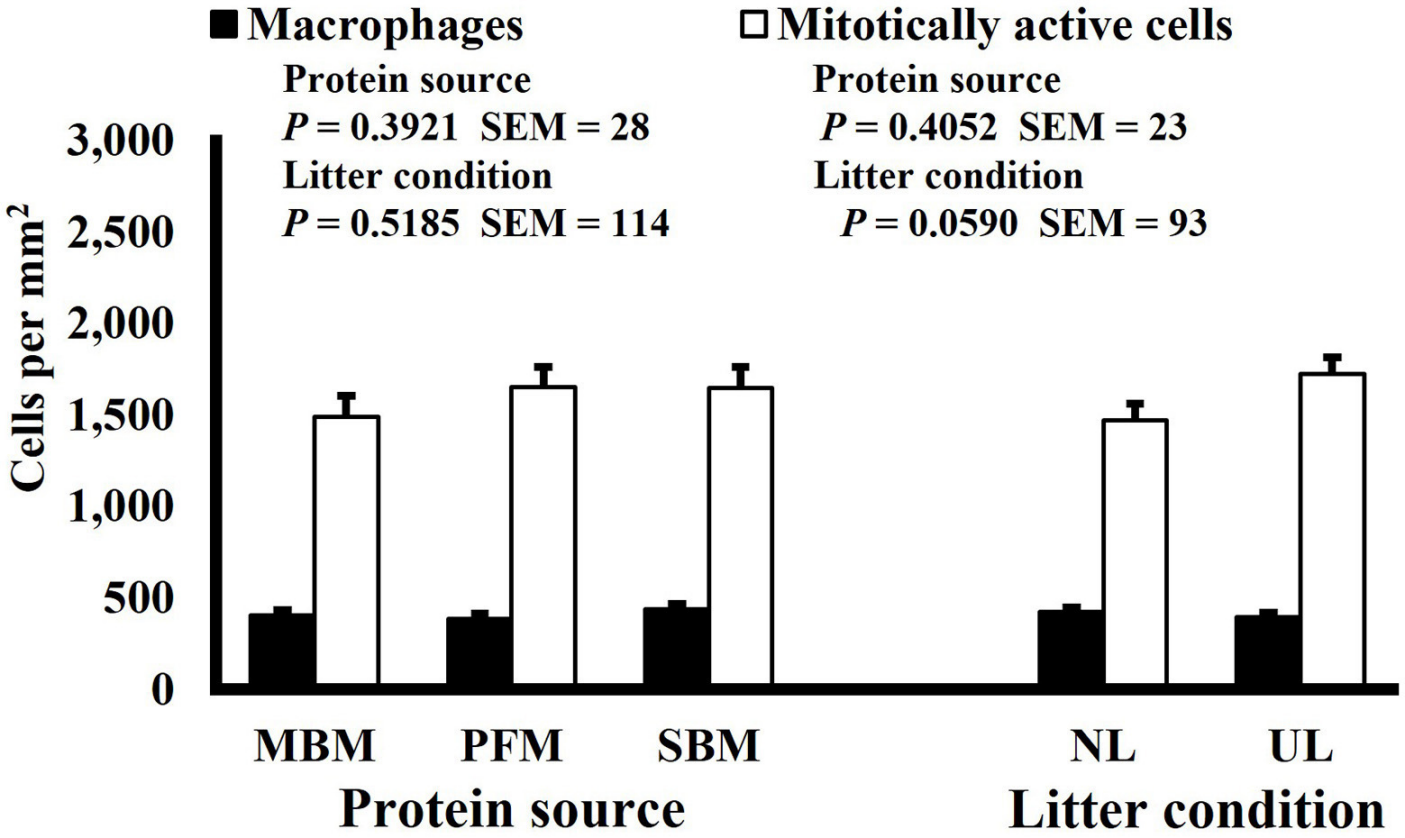
Effect of dietary protein source and litter condition on macrophage (KUL01+) and mitotically active (BrdU+) cell densities (cells per mm^2^) in the ileum of 8-d-old broilers (*n* = 36 per sampling d, 6 birds per treatment). MBM, meat and bone meal; PFM, 50% poultry by-product and 50% feather meal blend; SBM, soybean meal; NL, new litter; UL, used litter.

**Figure 8:**
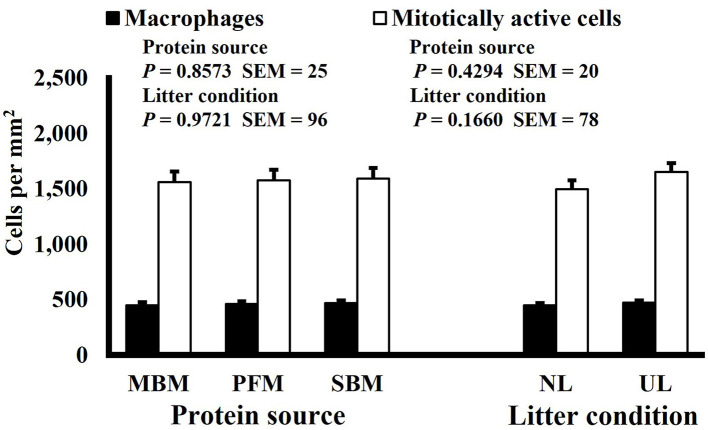
Effect of dietary protein source and litter condition on macrophage (KUL01+) and mitotically active (BrdU+) cell densities (cells per mm^2^) in the ileum of 11-d-old broilers (*n* = 36 per sampling d, 6 birds per treatment). MBM, meat and bone meal; PFM, 50% poultry by-product and 50% feather meal blend; SBM, soybean meal; NL, new litter, UL, used litter.

**Figure 9:**
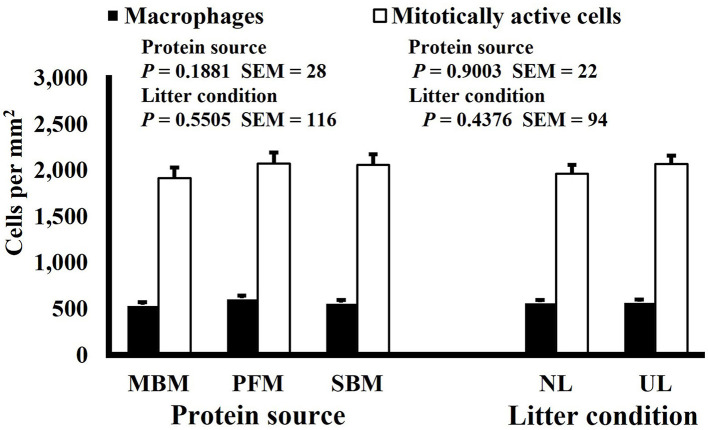
Effect of dietary protein source and litter condition on macrophage (KUL01+) and mitotically active (BrdU+) cell densities (cells per mm^2^) in the ileum of 15-d-old broilers (*n* = 36 per sampling d, 6 birds per treatment). MBM, meat and bone meal; PFM, 50% poultry by-product and 50% feather meal blend; SBM, soybean meal; NL, new litter; UL, used litter.

**Figure 10:**
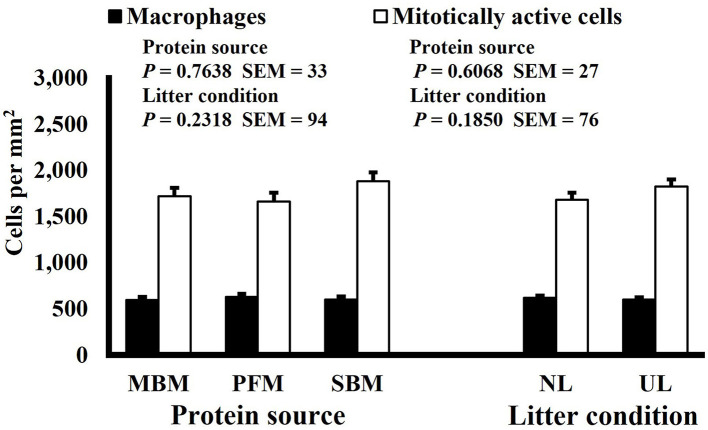
Effect of dietary protein source and litter condition on macrophage (KUL01+) and mitotically active (BrdU+) cell densities (cells per mm^2^) in the duodenum of 8-d-old broilers (*n* = 36 per sampling d, 6 birds per treatment). MBM, meat and bone meal; PFM, 50% poultry by-product and 50% feather meal blend; SBM, soybean meal; NL, new litter; UL, used litter. ^a, b^Bars of the same color representing means from either dietary protein source or litter condition with different superscripts differ at *P* ≤ 0.05.

**Figure 11:**
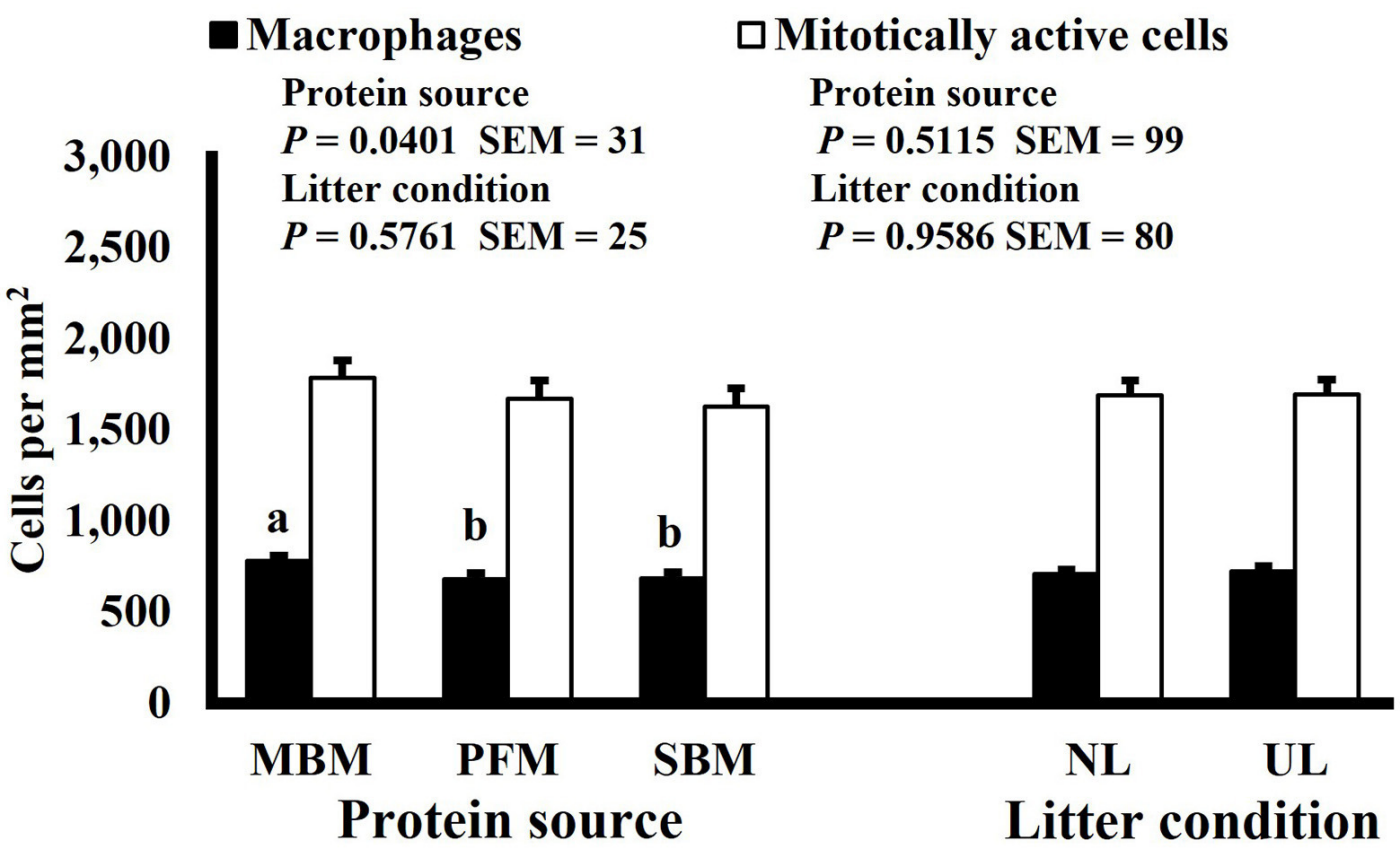
Effect of bird age on macrophage (KUL01+) and mitotically active (BrdU+) cell densities (cells per mm^2^) in the duodenum of broilers (*n* = 36 per d-of-age). ^a−d^ and ^A−D^Bars of the same color with different superscripts differ at *P* ≤ 0.05.

Neither dietary protein or litter condition altered mitotically active cell or macrophage densities on d 11 and 21 in the duodenum (*P* ≥ 0.1016; Figures 5, 6, respectively) or on d 3, 8, 11, and 15 in the ileum (*P* ≥ 0.059; Figures 7–10, respectively). Though macrophage density was greater in the duodenum of broilers fed MBM on d 8 (781 vs. 682 and 686 macrophages per mm^2^, MBM, PFM, and SBM, respectively; *P* ≤ 0.0401; Figure 11). An increase in local macrophage density could be the result of a local, mild immune response which would recruit proinflammatory macrophages to the area. There is some evidence suggesting that diets containing high levels of glycine, such as those including MBM, support the proliferation of *Clostridium perfringens*, which is the primary causative agent of necrotic enteritis. Wilkie et al. established a positive correlation among dietary and ileal glycine content with *C. perfringens* numbers in the ileum and ceca in broilers orally inoculated with *C. perfringens* and fed a variety of protein sources (13). Necrotic enteritis lesions typically affect the lower portions of the small intestine, but lesions can occur in the duodenum (24). Dahiya et al. (25) found that in broilers orally challenged with C. *perfringens* higher inclusion of glycine in the diet increased the *C. perfringens* populations in the ceca, and they also noted the occurrence of duodenal lesions (25). Higher glycine content in the MBM diet could predispose broilers to necrotic enteritis and be the cause of the higher macrophage density observed in this study; however, this result was only observed at one timepoint and occurred in the duodenum, which can be, but is not typically affected by *C. perfringens*.

**Figure 12:**
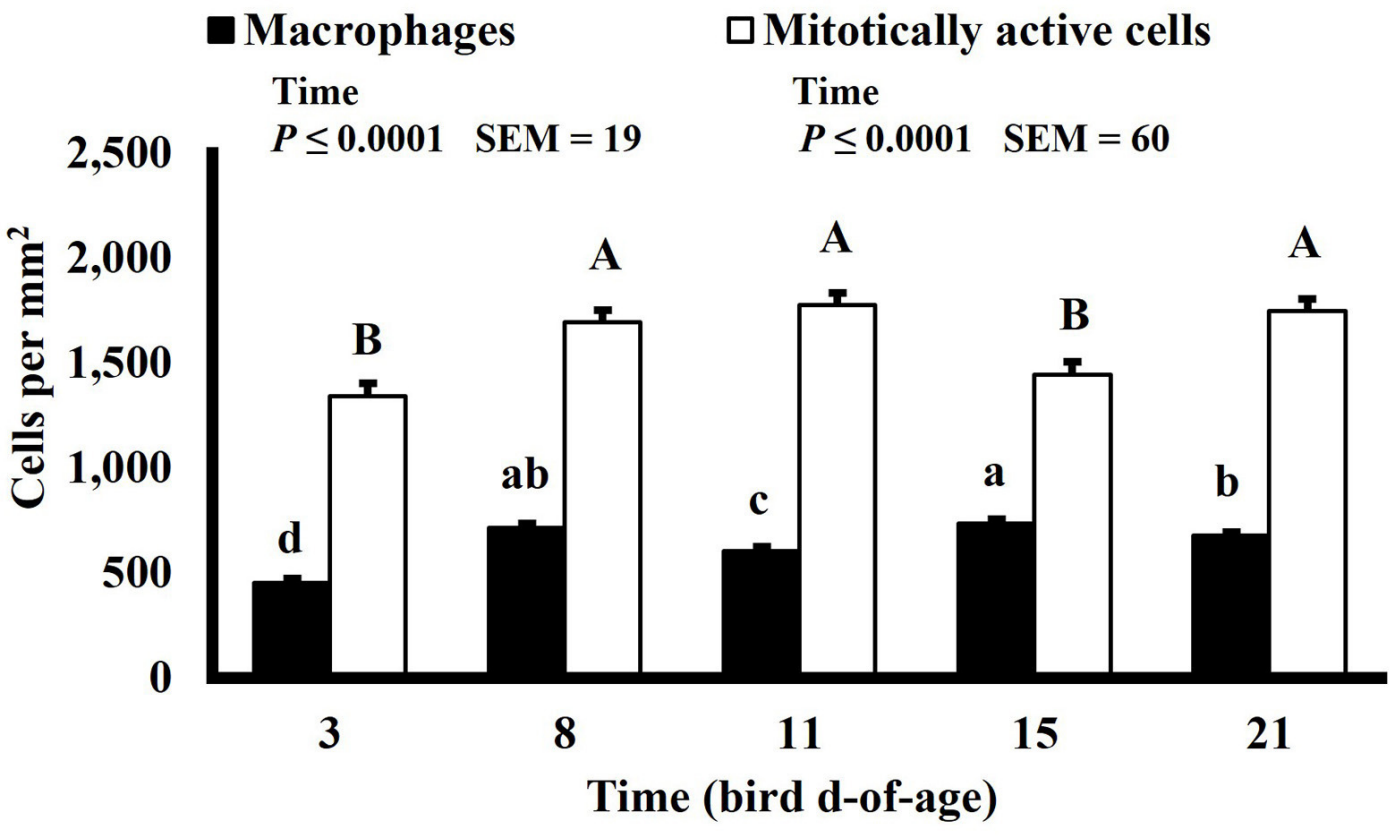
Effect of bird age on macrophage (KUL01+) and mitotically active (BrdU+) cell densities (cells per mm^2^) in the ileum of broilers (*n* = 36 per d-of-age). ^a−d^ and ^A−D^Bars of the same color with different superscripts differ at *P* ≤ 0.05.

**Figure 13:**
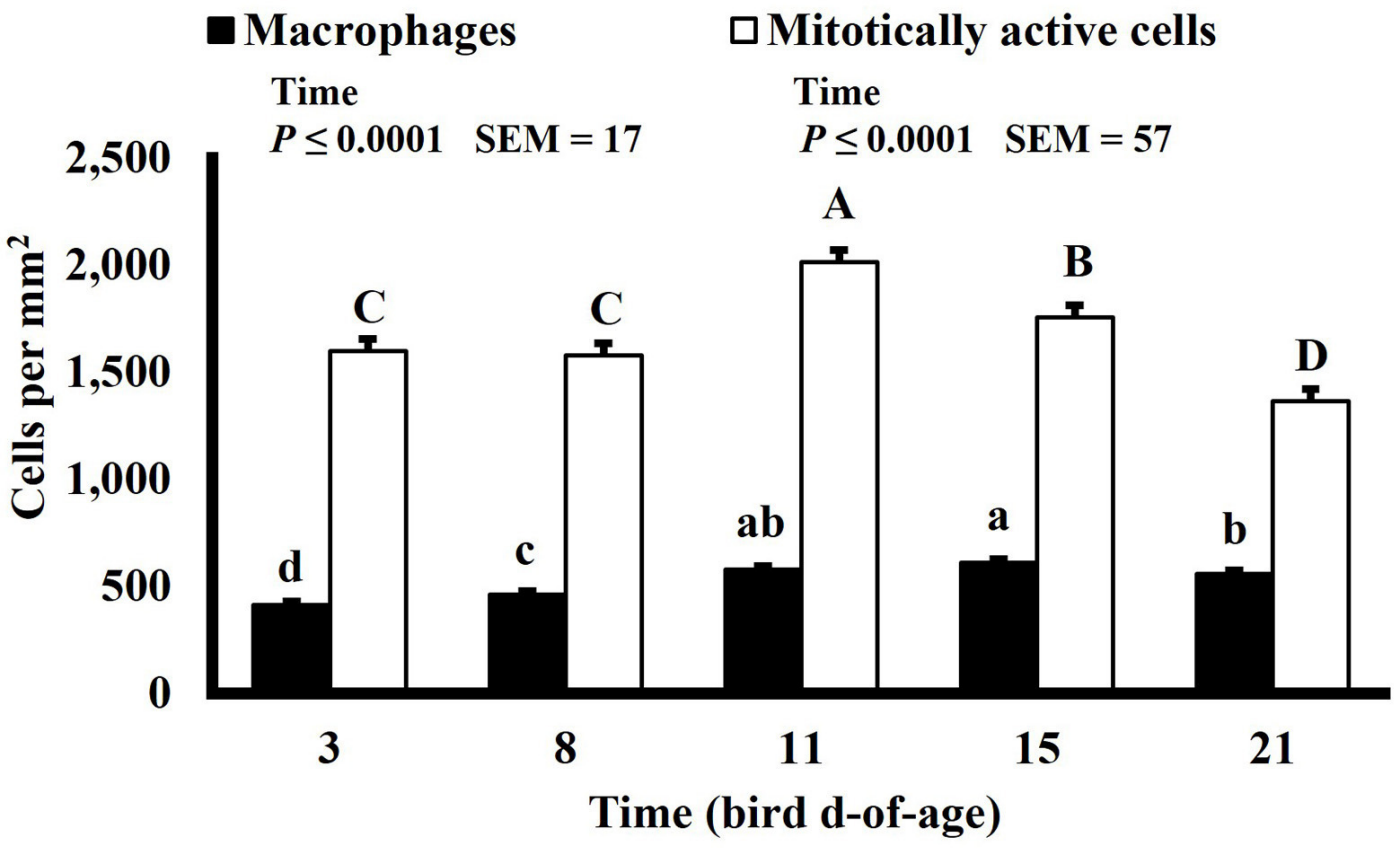
Representative digital images illustrating immunofluorescence detection of macrophages (KUL01+), including pro-inflammatory and anti-inflammatory macrophages, and mitotically active (proliferating; BrdU+) cells in duodenal (A,B) and ileal (C,D) cryosections of 21-d-old broilers. (A,C) Total macrophages (red, KUL01+). (B,D) Mitotically active cells (pink, BrdU+). Scale bar = 100 μm.

Mitotically active cell density changed in both the duodenum and ileum over time. Mitotically active cell density of the duodenum increased on d 8 to 1,694 mitotically active cells per mm^2^, decreased on d 15 to 1,444 mitotically active cells per mm^2^, and increased again on d 21 to 1,746 mitotically active cells per mm^2^ (*P* ≤ 0.0001; Figure 12), and in the ileum, mitotically active cell density increased to its highest value on d 11 at 2,016 mitotically active cells per mm^2^, decreased on d 15 to 1,755 mitotically active cells per mm^2^ and decreased again, to its lowest value of 1,364 mitotically active cells per mm^2^, on d 21 (*P* ≤ 0.0001; Figure 13). The GI tract of chicks quickly develops, both in maturity and overall size, post-hatch to facilitate rapid ability to digest and absorb nutrients from ingested feed, and this must be accomplished by mitotic activity. Geyra et al. described the development of chick enterocytes as occurring in 2 periods (26). During the first 24 h post-hatch, enterocytes differentiated from small, round cells lacking a well-defined brush border to the polar shape of mature enterocytes with a distinct brush border. During the second period, from d 3–6, the enterocytes in the duodenum increased in cell length; however ileal enterocytes did not undergo significant morphological change post-hatch. In a study by Iji et al. it was observed that duodenum and ileum length increased until d 7 but not after d 14, with similar results of villus surface area (27). Therefore, the timepoints selected in this study likely did not capture the early development of the intestine and changes observed as the birds aged may be in response to some type of environmental stimuli rather than efforts to increase the overall volume of intestinal mucosa.

Macrophage density was lowest on d 3 in the duodenum (453 macrophages per mm^2^) and followed a pattern of increasing and decreasing with each timepoint thereafter (*P* ≤ 0.0001; Figure 12). In the ileum, macrophage density was also lowest on d 3 at 411 macrophages per mm^2^ but increased until d 15 to 610 macrophages per mm^2^ and decreased on d 21 to 558 macrophages per mm^2^ (*P* ≤ 0.0001; Figure 13). There is a lack of literature discussing changes in macrophage density in the intestines of a growing and developing animal. Therefore, it is possible that the changes in macrophage density observed over time represent changes that typically occur rather than an influx of macrophages in response to some type of enteric insult. This could be further studied by differentiating pro and anti-inflammatory macrophages as intestinal resident macrophages express an anti-inflammatory profile while macrophages recruited to the intestines during an immune response express a proinflammatory profile (28).

In conclusion, this study establishes an experimental model to evaluate 2 important GIT and local immune developmental parameters while simultaneously evaluating how dietary protein source and the condition of the litter influence these parameters in an ABF environment. Ultimately, increased mitotically active cell density observed in broilers reared on UL and increased macrophage density in broiler fed MBM represent a use of energy and amino acids, which must be supplied through the diet, not directly utilized to support lean muscle growth. As producing lean meat products is the primary goal of the broiler industry, the underlying mechanisms producing these results warrants future study. This model will allow these questions, along with others related to ABF broiler production and enteric diseases, to be further explored in an effort to continue improving the efficiency and sustainability of animal agriculture.

## Data Availability Statement

The original contributions presented in the study are included in the article/Supplementary Material, further inquiries can be directed to the corresponding author/s.

## Ethics Statement

The animal study was reviewed and approved by Auburn University Institutional Animal Care and Use Committee.

## Author Contributions

AK, AC, OT, CS, and JS conducted the experiments, analyzed samples, and collected data. AK, CS, and JS analyzed the data. AK wrote the original manuscript draft. JS and CS oversaw all experiments and revised the manuscript. All authors contributed to the article and approved the submitted version.

## Funding

The study was supported by the United States Department of Agriculture National Institute of Food and Agriculture (USDA-NIFA) through Hatch Act funds to the Alabama Agricultural Experiment Station.

## Conflict of Interest

The authors declare that the research was conducted in the absence of any commercial or financial relationships that could be construed as a potential conflict of interest.

## Publisher's Note

All claims expressed in this article are solely those of the authors and do not necessarily represent those of their affiliated organizations, or those of the publisher, the editors and the reviewers. Any product that may be evaluated in this article, or claim that may be made by its manufacturer, is not guaranteed or endorsed by the publisher.
