# Supplementary material for: Dietary Protein Source and Litter Condition Alter Broiler Chicken Intestinal Macrophage and Mitotically Active Cell Populations
Source: Front Vet Sci. 2022 Apr 13;9:894587. doi: 10.3389/fvets.2022.894587 (PMC9043855; doi:10.3389/fvets.2022.894587)
Supplement: Supplementary file 1 [file Data_Sheet_1.PDF]

Supplementary Material

Supplementary Table 1. Effect of dietary protein source and litter condition on growth performance of broilers reared to 21 d-of-age.

	Dietary protein source					Litter condition			
Variable	MBM[4]	PFM[5]	SBM[6]	SEM	PROT *P*-value	NL[7]	UL[8]	LITTER *P*-value	PROT × LITTER *P*-value
BWG[1], g	845	869	821	23	0.3427	848	842	0.8341	0.4994
FI[2], g	1,100	1,077	1,057	31	0.6333	1,079	1,077	0.9524	0.6241
FCR[3]	1.3008[a]	1.2392[b]	1.2874[a]	0.01	0.0002	1.2735	1.2781	0.6725	0.3659

[1]BWG = body weight gain
[2]FI = feed intake
[3]FCR = feed conversion ratio (FI:BWG)
[4]MBM = porcine meat and bone meal; (n = 100 pens)
[5]PFM = 50% poultry by-product and 50% feather meal (n = 100 pens)
[6]SBM = soybean meal (n = 100 pens)
[7]NL = new litter (n = 150 pens)
[8]UL = used litter (n = 150 pens)
[a,b]Means within a row within a dietary protein source or litter condition with different superscripts differ $P \leq 0.05$
